# Child Neuropsychological Functioning and Interpersonal Callousness as Predictors of Externalising Behaviour in Early Adolescence: A Prospective Population-based Study

**DOI:** 10.1007/s10802-023-01091-8

**Published:** 2023-06-08

**Authors:** Isabel E. de Graaf, Koen Bolhuis, Charlotte A. M. Cecil, Tonya H. White, Josanne D. M. van Dongen

**Affiliations:** 1 Department of Psychology, Education and Child Studies, Erasmus University Rotterdam, Burg. Oudlaan 50, 3062 Rotterdam, PA the Netherlands; 2 Department of Child and Adolescent Psychiatry/Psychology, Erasmus MC, Rotterdam, the Netherlands; 3 Department of Epidemiology, Erasmus MC, Rotterdam, the Netherlands; 4 Molecular Epidemiology, Department of Biomedical Data Sciences, Leiden University Medical Center, Leiden, the Netherlands; 5 Department of Radiology and Nuclear Medicine, Erasmus MC, Rotterdam, the Netherlands; 6 Section of Social and Cognitive Developmental Neuroscience, National Institutes of Mental Health, Bethesda, MD USA

**Keywords:** Neuropsychological functioning, Externalizing, Aggression, Callous traits, Moderator

## Abstract

Externalizing problems are a key predictor of individual functioning in adulthood. Therefore, identifying possible risk factors for externalising problems is valuable for optimising prevention and treatment programmes. Previous research has shown that (domains of) neuropsychological functioning predict externalising problems later in life. However, the influence of callous traits, and sex as potential moderators in this relation remains unclear. The aim of this study was to examine associations between neuropsychological functioning in children (at age 8 years) and later externalising behaviour in adolescence (at age 14 years), as well as to test the role of callous traits (at age 10 years) and sex as moderating factors. The analyses were conducted using data from 661 Dutch children from the population-based Generation R Study (47.2% female). We found no association between neuropsychological functioning and later externalising behaviour. However, callous traits predicted externalising problems at age 14 years. Further, callous traits moderated the association between neuropsychological functioning and externalising behaviour, though this association dropped below the statistical significance level when adjusted for confounders. Specifically, while higher neuropsychological functioning was associated with more externalising behaviour in children with high callous traits, lower neuropsychological functioning was not associated with externalising behaviour in children with low callous traits. Although boys showed significantly higher externalising behaviours compared to girls, no moderating effect of sex was found on the association between neuropsychological functioning and externalising behaviour. These results add to a growing body of evidence supporting a distinct neurocognitive profile in children with high vs low callousness.

**Supplementary Information:**

The online version contains supplementary material available at 10.1007/s10802-023-01091-8.

## Introduction

Externalising problems in childhood, including aggression, conduct problems, oppositional defiant behaviour, and delinquency are associated with a variety of negative outcomes in adolescence and adulthood (Campbell et al., 2000). For example, aggression at a young age is associated with a high financial burden on society due to increased risk for criminality, unemployment, and public service usage, as well as substance use and mental health problems later in life (Brook et al., 1992; Foster & Jones, 2005; Rivenbark et al., 2018). Consequently, it is essential to detect risk for child externalising problems early and to implement timely interventions. The idea is that by the early identification of risk factors for externalising behaviour, successful prevention programmes can be developed and implemented, which in turn can forestall the development of more aggravating problems such as development of an antisocial personality disorder.

### Neuropsychological Functioning as a Risk Factor

One factor that has received substantial attention in relation to externalising problems is neuropsychological functioning (Frick et al., 2014; Rohlf et al., 2018; Thompson et al., 2019). neuropsychological functioning includes a diverse set of abilities including language, learning and memory, social cognition, perceptual-motor function, and executive functions (Séguin & Zelazo, 2005; Thomson & Centifanti, 2018). Several independent studies have reported an association between poorer executive functioning – defined as higher-order cognitive abilities, such as attention, planning, abstract reasoning, inhibitory control and working memory (Jurado & Roselli, 2007; Morgan & Lilienfeld, 2000) – and various forms of externalising behaviour, such as delinquency, criminality and physical aggression (Morgan & Lilienfeld, 2000; Ogilvie et al., 2011). However, potential associations with other neurocognitive domains remain unclear. For example, a recent meta-analysis found that low language ability predicts the later development of externalising behaviour in different samples of school-aged children that differed in risk status and age (Chow et al., 2018). In contrast, a large population-based study including children from the general population found no associations between externalising behaviour at age 6 years (Blanken et al., 2017) and language abilities at age 8 years, although it did confirm an association with poor attention and EF. Further, very few studies have examined both global neuropsychological functioning and different subsets, so that it is unclear whether previously identified associations are domain specific or generalized. Indeed, a recent large longitudinal study of neurodevelopment (The Adolescent Brain Cognitive Development Study) in children from the general population of age 8 and 9 years, found that externalising behaviour was predicted by lower levels of general cognitive ability, not by EF specifically (Thompson et al., 2019).

### Callousness and Externalising Behaviour

In addition to low neuropsychological functioning, callous-unemotional traits are also found to be an important predictor of later externalising behaviour (Frick et al., 2005). Callous traits involve characteristics such as being mean to others and not feeling guilty about one's own actions, which are elements of a more extensive set of callous-unemotional traits (CU-traits) and psychopathic traits (Frick, 2004). The DSM-V, which refers to CU-traits as 'limited prosocial emotions', has included this behaviour as a specifier for conduct problems (APA, 2013). CU-traits involve, next to callous traits, shallow emotions, and disregard for the feelings of others (Frick, 2009). Numerous different measures, varying in their inclusion of aforementioned specific behaviours, have been used to study callous-unemotional traits. This needs to be considered when interpreting the existing literature.

Previous research has shown that in a sample of children from the general population, CU-traits at age 3 years predict externalising problems at later ages, namely age 6 years and 10 years, over and above earlier externalizing problems at age 3 years (Song et al., 2016). Additionally, a systematic review including eight different samples from different populations (e.g. general population, clinically referred, detained) and from different age levels (ranging between 5 and 18 years of age), that children with conduct problems and CU-traits are more at risk for severe externalising behaviour later in life than children with CD without CU-traits (Colins et al., 2020).

When looking at the relation between neuropsychological functioning and CU-traits, it has been reported that high CU-traits and high EF predicted greater violent behaviour in a high-risk sample of adolescents (Baskin-Sommers et al., 2015). A more recent population-based study that looked at both intelligence and executive functioning, found CU-traits to be a moderator for the association between sustained attention specifically, and antisocial behaviour in a sample of adolescents (mean age 14 years) from the general population (Dotterer et al., 2021). At high levels of CU-traits, antisocial behaviour in children was associated with better performance on a sustained attention task, whereas it was associated with worse performance with low levels of CU-traits. In contrast to these results, Waller et al. (2017) found that CU-traits at age 3 predict aggression in late childhood and that *low levels of EF* modulated this effect. These results imply that CU-traits could be a possible moderator of the association between neuropsychological functioning and externalising behaviour, although the findings are mixed.

Mixed evidence about the role of neuropsychological functioning and CU traits in externalising problems could be due to substantial differences in study design (cross-sectional vs longitudinal), timing of variables assessed (childhood vs adolescence) and samples used (e.g. community-based vs offender and high-risk clinical samples). Prospective studies using well-characterized, longitudinal data across development are needed to better understand the associations between EF, CU-traits and externalising behaviour in children. Furthermore, the results from studies that included offenders or high-risk samples may not generalise to the general population. The latter implies that interventions based on the data of offender samples may not be as effective in non-offending children.

### Sex Differences and Externalising Behaviour

Sex could also be important in understanding the association between neurocognitive functioning and externalising behaviour due to sex differences in the occurrence and manifestation of externalising behaviours. For example, it is well-established in the literature that boys show more externalising problems than girls (Arnett et al., 2014; Mayes et al., 2020), and score higher on CU- traits than girls (Essau et al., 2006; Pihet et al., 2015). Further, some studies have shown that sex moderates the association between neurocognitive functioning and externalising behaviours. For example, one study found that the negative association between EF (inhibition) and (proactive) aggression was stronger and only significant for girls (Granvald & Marciszko, 2015). However, this moderating effect was not found for reactive aggression or other forms of aggression in relation to poor EF (McQuade et al., 2013; White et al., 2013b). Thus, it remains unclear whether sex moderates the effect of neuropsychological functioning on externalising behaviour in children.

In summary, although previous research has examined the relation between neuropsychological functioning and externalising behaviour later in life, key gaps in the literature remain. First, previous studies looking into the role of global neuropsychological functioning vs subdomains of neuropsychological functioning are lacking. We want to look at the associations between neuropsychological functioning more comprehensively to disentangle general vs specific associations between neuropsychological functioning and externalising behaviour. Secondly, the moderating role of CU-traits, and especially interpersonal callousness in the association between neuropsychological functioning and externalising behaviour remains unclear. Third, studies have been mainly being carried out in boys and not in girls, so there is a need for a sex- balanced study to examine potential sex-related differences and moderating effects. Last, most studies have relied on cross-sectional data from offender or high-risk samples; as such, the extent to which previously identified associations generalize to dimensional symptoms in the general population.

Further, the use of data from large, prospective longitudinal studies is needed to clarify the temporal sequence of the association between child neuropsychological functioning and externalizing in adolescence. This is of great importance because it could expand our knowledge on why certain children develop externalising behaviours and others do not. If this is known, prevention strategies could be adapted and optimised.

### Present Study

This study aimed to clarify the role of child neuropsychological functioning as a predictor of later externalising behavioural problems in a large, population-based sample, as well as testing the potential moderating role of callous traits and sex. Using longitudinal data from a large, population-based study, we investigated (a) whether global neuropsychological functioning and its individual domains measured in middle childhood (age 8yrs) prospectively associate with externalising behaviour in emerging adolescence (age 14yrs), and (b) whether callous traits (10 yrs) and sex moderate these relationships. Analyses were adjusted for a range of covariates, as well as baseline externalising behaviour and co-occurring psychopathology to establish the unique links between neuropsychological functioning, callous traits, and later externalising behaviour (the Generation R Study; Kooijman et al., 2016).

We hypothesized that lower neuropsychological functioning in mid childhood would uniquely predict higher externalising behaviour at age 14 over and above existing (i.e. baseline) levels of externalising problems. Second, when looking at the different tasks used to measure neuropsychological functioning, it was expected that tasks indexing EF would not significantly predict externalising behaviour over and above other measured domains, because some studies show multiple domains to be associated with externalising behaviour (Thompson et al., 2019). Third, it was hypothesised that callous traits at the age of 10 years would predict externalising problems at age 14 years (Rizeq et al., 2020). Fourth, based on growing evidence about distinct neurocognitive profiles associated with high- vs low- levels of CU-traits (Dotterer et al., 2021), it is expected that callous traits will significantly moderate the relationship between neuropsychological functioning and externalising problems. Fifth, given that previous studies have focused mainly on males, we expected boys to show more callous traits and externalising, but no a priori hypotheses were formulated regarding sex as a moderator.

## Method

### Participants

The present study is based on data from the Generation R Study, a population-based prospective cohort study (Kooijman et al., 2016), which included pregnant women in Rotterdam, the Netherlands, from 2001–2005. In total, 9778 mothers with a delivery date from April 2002 until January 2006 were enrolled in the study. Data collection of the children started during foetal life and is ongoing. The study is designed to identify early causes leading to normal and abnormal development and health in children throughout their lives. Participants are currently undergoing the 17–19-year assessment wave. The Generation R Study is approved by the Medical Ethical Committee of the Erasmus Medical Center, Rotterdam, the Netherlands.

Children who had data on the measures for neuropsychological functioning, callousness and externalising behaviour were eligible for further analysis. The data used in this study was collected at an average age of 8, 10 and 14 years. Of the 1307 participants who were included in the sub-study on brain imaging and neuropsychological functioning (i.e. where neuropsychological functioning was assessed), 929 participants had data available on externalising problems at mean age 14 years. Descriptive characteristics and percentage of missing data are shown in Table 1. Non-response analyses showed that mothers of children included in this study were higher educated than mothers of non-included children, and they also showed less depression, anxiety, and hostility. Additionally, included children had often a Dutch nationality or a Non-Dutch Western nationality and were younger than excluded children during most data collection moments, except during the assessment of neuropsychological functioning (p < 0.05).

**Table 1:** Descriptive characteristics of the sample (*N* = 929 participants)

**Variable**	**Statistic**	**Missing (%)**
*Child characteristics*		
Age at CBCL (6) in years, mean (SD)	6.01 (0.39)	50 (5.38%)
Age at NEPSY-II-NL in years, mean (SD)	7.94 (0.99)	0
Age at callousness in years, mean (SD)	9.84 (0.38)	239 (25.73%0
Age at CBCL (13) in years, mean (SD)	13.56 (0.40)	0
Sex, number (%)		
Male	455 (48.98%)	0
Female	474 (51.02%)	0
Ethnicity, number (%)		7 (0.75%)
Dutch	682 (73.97%)	
Other, Western	66 (7.16%)	
Other, non-Western	174 (18.87%)	
Mother-reported CBCL (6), median (IQR)		
Externalising problems	8.00 (8.17)	54 (5.81%)
Internalising problems	6.00 (7.11)	52 (5.6%)
Neuropsychological functioning (8), median (IQR)		
Total score	0.27 (1.17)	0
Attention and executive functioning	0.26 (1.02)	0
Language	0.01 (1.36)	0
Memory and learning	0.14 (1.21)	0
Visuospatial	0.26 (1.19)	0
Sensorimotor	0.23 (1.15)	0
Mother-reported callousness (10), median (IQR)	2.00 (3.00)	250 (26.91%)
Mother-reported CBCL (13), median (IQR)		
Externalising problems	3.00 (7.00)	0
Aggressive behaviour	2.00 (6.00)	0
Internalising problems	5.00 (7.00)	0
*Maternal characteristics*		
Educational level, number (%)		58 (6.24%)
High	518 (59.88%)	
Medium	329 (38.03%)	
Low	18 (2.08%)	
Maternal psychopathology, median (IQR)		
Interpersonal sensitivity	0.25 (0.50)	112 (12.06%)
Depression	0.00 (0.33)	111 (11.95%)
Anxiety	0.17 (0.33)	112 (12.06%)
Hostility	0.20 (0.40)	111 (11.95%)

### Measures

#### Neuropsychological Functioning (Age 8)

Neuropsychological functioning was assessed using a shortened version of the Developmental Neuropsychological Assessment (NEPSY-II-NL; Korkman et al., 2010). The NEPSY- II-NL is a Dutch translation of the North American NEPSY-II and measures neuropsychological functioning in children aged 5- 12 years (Brooks et al., 2009). Acceptable to good test–retest reliability has been reported for the NEPSY-II; most of the tasks having a reliability coefficient of *r* > 0.70 (Brooks et al., 2009). The total test battery consists of 34 tasks, but due to time constraints, a selection of ten tasks was made: (1) Auditory attention and response set, (2) Statue, (3) Word generation, (4) Memory for faces, (6) Memory for faces delayed, (6) Narrative memory, (7), Visuomotor precision, (8) Arrows, (9) Geometric puzzles, (10) Route finding (White et al., 2013a). The tasks were administered by trained research assistants. Previous studies using this measure in the Generation R Study found through principal component analysis that the following theoretically derived domains are covered in the shortened version of the NEPSY-II-NL: (1) attention/executive functioning, (2) language, (3) memory and learning, (4) sensorimotor functioning, and (5) visuospatial processing (Mous et al., 2017; Blanken et al., 2017).

#### Callousness (Age 10)

Callousness was assessed at age 10 through maternal report using the adapted version of Pardini's Interpersonal Callousness scale (IC scale; Pardini et al., 2006) and Frick's Inventory of Callous-Unemotional Traits (ICU; Frick, 2004). This adapted brief questionnaire consists of seven statements regarding the child: (1) ‘Cannot be trusted with regard to what he/she says’, (2) ‘Denies having done something wrong, even though it is certain he/she did something wrong’, (3) ‘Uses or misleads other people in order to get what he/she wants, (4) ‘If confronted about his/her behaviour, he/she is able to talk himself/herself out of it easily’, (5) Does not keep any promises’, (6) ‘Does not find other people’s feeling important’ and (7) ‘Is cold and indifferent’. The behavioural statements are rated on a 4-point scale (range 0–21): 0 *Does not apply at all*, 1 *Does apply slightly*, 2 *Does apply very much*, 3 *Does apply completely*. Even though this measure does not capture all callous-unemotional traits, it has been shown to assess childhood callousness adequately on a dimensional scale (Pardini et al., 2006). Furthermore, Bolhuis et al. (2019) showed acceptable reliability of this scale in the Generation R Study (*α* = 0.73). The Cronbach’s α in this sample is 0.78. Endorsement of the seven items is shown in Supplemental Table S1.

#### Externalising Behaviour (Age 6 and 14)

The Child Behavioural Checklist (CBCL; Achenbach & Rescorla, 2000, 2001) for age 1,5 to 5 years (CBCL/1 -5) and for age 6 to 18 years (CBCL/6–18) was used to obtain reports completed by the primary caregiver on their children's externalising and internalising behaviour at age 6 and age 14 respectively. The primary caregivers (primarily mothers) indicated whether their child displayed any of the 99 behaviours as described in the CBCL in the past two months. Behavioural statements vary from '*Screams a lot*' to '*Selfish or won't share*' and were rated on a 3-point scale (0 *not true*, 1 *sometimes/somewhat true*, 3 *very/often true*), with higher scores indicating more severe problems. Various studies have supported the validity of the Dutch version of the CBCL (Verhulst et al., 1985). The CBCL yields eight subscales with good reliability and validity (Achenbach, 1991). These subscales also yield two broadband scales: Internalising problems (Withdrawn, Somatic Complaints, Anxious/Depressed) and Externalising problems (Aggressive behaviour and Rule-breaking) with both good reliability scores (*α* = 0.91 and *α* = 0.92, respectively).

#### Covariates

Several covariates were considered a priori. Child sex and age were used as covariates and were retrieved from birth records. Child ethnicity defined using the definition of Statistics Netherlands (CBS): (1) Dutch, (2) Other western, (3) Non-Dutch, Non-Western. Maternal education was categorised using three levels: low (no or primary education), middle (lower and intermediate vocational training), and high education (higher vocational training and university).

Further, to account for rater bias, we adjusted for maternal psychopathology (age 10) (Najman et al., 2001). The Brief Symptom Inventory is a self-report instrument consisting of 53 items (Derogatis & Melisaratos, 1983). For the current study, the following four subscales were used: interpersonal sensitivity, depression, anxiety, and hostility.

Because this study had the aim to predict later externalising behaviour over and above existing externalising problems, the outcomes were additionally adjusted for externalising problems at age 6 (baseline externalising behaviour). Also, since this research focuses on the contribution of neuropsychological functioning and callousness on externalising behaviour specifically, analyses were further controlled for co-occurring psychopathology (internalising problems) at age 14 years.

### Statistical Analyses

All analyses were performed in R version 3.6. Variables (more specifically, the CBCL externalising, internalising, and aggressive behaviour problems as well as the callous traits score) that showed moderate positive skew were square root transformed to approach normality. Missing data on covariates have been dealt with using multiple imputation using the *mice* package in R with 10 imputed datasets and 10 iterations. Imputed datasets were pooled for the regression analyses. Results on the imputed data were compared with the non-imputed complete case data, which were virtually unchanged (Supplemental Material, Tables S1-5).

First, descriptive characteristics and correlations between all study variables were calculated. Next, five regression analyses were run. In regression 1, a multiple linear regression analysis was conducted using the total score on the NEPSY-II-NL (i.e. neuropsychological functioning) at age 8 years to predict externalising behaviour at age 14 years. This model was adjusted for baseline externalising problems (age 6), callous traits and all covariates. To test the specificity of associations to externalising problems, the model was also adjusted for internalising problems at age 14 years. In a second regression, multiple linear regression analyses were conducted using different domain scores of the NEPSY-II- NL as simultaneous predictors of externalising behaviour at age 14, adjusting for the same covariates as regression 1. This was done to test whether EF uniquely and significantly predicted externalising behaviour over and above other neuropsychological functioning domains. In regression 3, we examined whether callous traits moderated the association between neuropsychological functioning and subsequent externalising problems. In regression 4, a linear regression analysis was conducted to use neuropsychological functioning at age 8 to predict externalising behaviour at age 14, testing the effects of sex as a moderator. Lastly, a three-way-interaction was examined, i.e. callous traits and sex were both used in a linear regression analysis to see whether the interaction among neuropsychological functioning and callous traits is different across sex.

Graphic visualisations of the interaction effects were performed in R, using the *ggplot2* package. In this interaction graph, the association between neuropsychological functioning at age 8 years (x-axis, i.e., the exposure) and externalising problems at age 14 years (y-axis, i.e. the outcome) was plotted separately for children with high versus low callous traits scores. Callous traits scores were dichotomised based on the 93^rd^ percentile (n = 43 in the high scoring group). Further, externalising problems scores were square root transformed as in the regression models, and both neuropsychological functioning and externalising problems scores were standardised with a mean of zero and a standard deviation of one for comparability.

## Results

Descriptive statistics are displayed in Table 1 and intercorrelations between variables are shown in Table 2.

**Table 2:** Correlations between all study variables

	1	2	3	4	5	6	7	8	9	10	11	12	13	14	15	16	17	18	19
1. NEPSY total (8)																			
2. Attention & executive functioning (8)	0.87*																		
3. Language (8)	0.59*	0.32*																	
4. Memory & learning (8)	0.72*	0.38*	0.47*																
5. Visuo-spatial (8)	0.60*	0.24*	0.32*	0.43*															
6. Sensorimotor (8)	0.48*	0.32*	0.28*	0.31*	0.30*														
7. Externalising problems (6)	-0.07*	-0.09*	-0.04	-0.02	0.02	-0.05*													
8. Internalising problems (6)	-0.16*	-0.14*	-0.12*	-0.13*	-0.05*	-0.03	0.71*												
9. Callous traits (10)	-0.05	-0.06*	0.00	-0.02	-0.01	-0.07	0.42*	0.32*											
10. Externalising problems (13)	-0.05	-0.06*	-0.01	0.00	-0.01	-0.08	0.54*	0.39*	0.44*										
11. Internalising problems (13)	-0.06*	-0.07	-0.01	-0.05	0.00	-0.06	0.33*	0.45*	0.26*	0.55*									
12. Aggressive behaviour (13)	-0.04	-0.06*	-0.02	-0.01	-0.01	-0.07	0.54*	0.40*	0.41*	0..98*	0.56*								
13. Sex	0.07*	0.11*	0.05	0.08*	-0.19*	0.17*	-0.18*	-0.08*	-0.12*	-0.17*	0.01	-0.13*							
14. Age child (13)	0.12	0.10	0.05	0.09	0.02	0.14*	0.04	0.01	-0.03	0.04	0.02	0.05	-0.01						
15. Child ethnicity	-0.13*	-0.09*	-0.23*	-0.07*	-0.14*	-0.07*	0.19*	0.20*	0.15*	0.13*	0.13*	0.13*	0.03	0.12*					
16. Maternal education level	-0.20*	-0.12*	-0.19*	-0.15*	-0.25*	-0.07*	0.05*	0.11*	0.08*	0.05*	0.10*	0.02	-0.04	0.00	0.12*				
17. Maternal interpersonal sensitivity	-0.07*	-0.04*	-0.04	-0.03	-0.04	-0.06*	0.14*	0.15*	0.13*	0.10*	0.19*	0.10*	-0.01	0.05	0.13*	0.02			
18. Maternal depression	-0.16*	-0.15*	-0.08*	-0.07*	-0.16*	-0.11*	0.17*	0.19*	0.15*	0.17*	0.28*	0.16*	0.00	0.07	0.17*	0.08*	0.70*		
19. Maternal anxiety	-0.02	-0.01	-0.05	0.01	-0.01	-0.06	0.16*	0.16*	0.10*	0.14*	0.19*	0.14*	0.01	0.04	0.10*	-0.02	0.55*	0.60*	
20. Maternal hostility	-0.02	-0.02	-0.01	0.02	-0.01	-0.06	0.25*	0.19*	0.20*	0.28*	0.26*	0.28*	0.02	0.06	0.12*	-0.02	0.56*	0.60*	0.54*

* denotes *P* < 0.05

Regression 1 (Table 3) showed that neuropsychological functioning was not associated with externalising problems at age 14, over and above covariates, baseline levels of externalising problems, and co-occurring internalising problems (with the latter two variables being the strongest predictors in the model, *β* = 0.29, *β* = 0.40, respectively). However, callous traits did significantly independently predict externalising behaviour at the age of 14 (*β* = 0.19, 95% CI 0.12–0.26). Overall, the model explained 51% of the variance.

**Table 3:** Regression analysis for the association between neuropsychological functioning and subsequent externalising problems in preadolescents

	β	*se*	95% CI	*t*	*P*
Neuropsychological functioning	0.01	0.03	-0.03; 0.06	0.54	0.590
Externalising behaviour (6)	0.29	0.03	0.23; 0.35	9.42	< 0.001**
Internalising behaviour (13)	0.40	0.03	0.34; 0.45	14.19	< 0.001**
Callousness (9)	0.19	0.03	0.12; 0.26	0.05	< 0.001**
Female sex	-0.14	0.05	-0.23; -0.04	-2.78	0.006**
Age (13)	0.03	0.02	-0.02; 0.08	1.14	0.255
Non-Dutch Western Ethnicity	-0.23	0.10	-0.42; -0.04	0.13	0.019*
Non-Western ethnicity	-0.16	0.07	-0.30; -0.03	-2.39	0.017*
Maternal education – medium	-0.05	0.06	-0.16; 0.06	-0.86	0.393
Maternal education low	-0.08	0.19	-0.45; 0.28	-0.45	0.655
Maternal interpersonal sensitivity	-0.02	0.04	-0.09; 0.06	-0.44	0.659
Maternal depression	-0.05	0.04	-0.13; 0.03	-1;35	0.179
Maternal anxiety	-0.04	0.03	-0.11; 0.02	-1.27	0.204
Maternal hostility	0.11	0.04	0.04; 0.18	3.14	0.002**

Presented are standardized coefficients (betas) and their 95% confidence intervals

*CI* confidence interval

R^2^ = 0.51, p = < 0.001

* significant for p < 0.05, ** significant for p = < 0.01

When examining separate NEPSY-II-NL domains in regression 2 (Table 4), we found that none of these domains were statistically significant independent predictors for externalising behaviour, over and above other variables in the model (all *p* > 0.05).

**Table 4:** Multiple regression analysis for the association between the subdomains of neuropsychological functioning and externalising problems in preadolescents

	β	*se*	95% CI	*t*	*P*
Attention and executive functioning	-0.04	0.03	-0.09; 0.02	-1.33	0.185
Language	0.03	0.03	-0.03; 0.09	1.10	0.271
Memory and learning	0.03	0.03	-0.03; 0.09	1.05	0.295
Visuo-spatial functioning	0.00	0.03	-0.07; 0.06	-0.15	0.880
Sensorimotor functioning	0.02	0.03	-0.04; 0.07	0.71	0.480
Externalising behaviour (6)	0.29	0.03	0.23; 0.35	9.23	< 0.001**
Internalising behaviour (13)	0.40	0.03	0.34; 0.45	14.18	< 0.001**
Callousness (9)	0.19	0.03	0.12; 0.26	5.62	< 0.001**
Female sex	-0.15	0.05	-0.25; -0.04	-2.80	0.005**
Age (13)	0.03	0.03	-0.02; 0.07	1.00	0.315
Non-Dutch Western Ethnicity	-0.21	0.10	-0.40; -0.02	-2.19	0.029*
Non-Western ethnicity	-0.15	0.07	-0.29; -0.02	-2.20	0.028*
Maternal education – medium	-0.04	0.06	-0.15; 0.07	-0.68	0.496
Maternal education low	-0.08	0.19	-0.45; 0.28	-0.44	0.663
Maternal interpersonal sensitivity	-0.02	0.04	-0.09; 0.06	-0.47	0.637
Maternal depression	-0.05	0.04	-0.14; 0.03	-1.34	0.182
Maternal anxiety	-0.04	0.04	-0.11; 0.03	-1.22	0.221
Maternal hostility	0.11	0.04	0.04; 0.18	3.18	0.002**

Presented are standardised coefficients (betas) and their 95% confidence intervals

*CI* confidence interval

R^2^ = 0.51, p = < 0.001

* significant for p < 0.05, ** significant for p = < 0.01

**Fig. 1:**
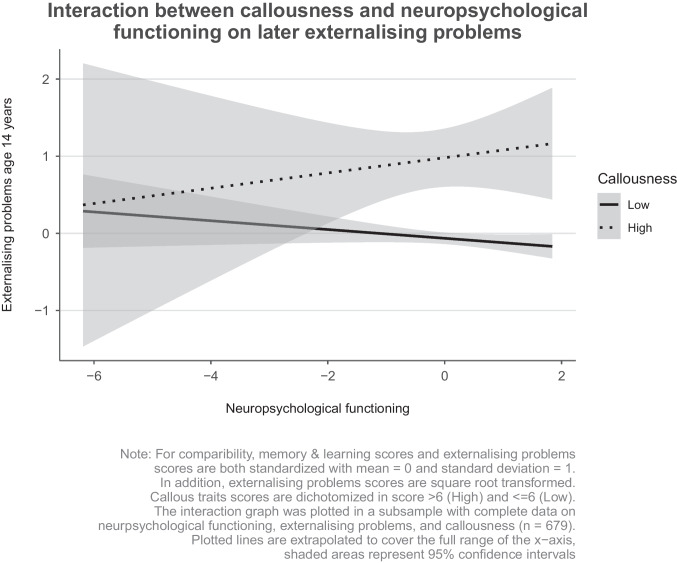
Visualization of interaction effects between total neuropsychological functioning and callousness

When testing for moderation effects (Table 5), we observed that callous traits moderated the association between neuropsychological functioning and later externalising problems, as can be seen by the non-overlapping confidence intervals in Fig. 1 (β_interaction_ = 0.06, 95% CI 0.00–0.12, *p* = 0.035). However, in the model fully adjusted for all covariates, baseline externalising problems and co-occurring internalising problems, this effect was no longer significant, although confidence intervals substantially overlapped with the non-adjusted association (β_interaction_ = 0.05, 95% CI 0.00–0.10, *p* = 0.062). As can be seen in Fig. 1, higher scores of neuropsychological functioning were marginally associated with higher scores of externalising problems, but only for children with high (i.e., score > 6, which is the 93^rd^ percentile) callous trait scores. This association was not observed for children with low callous traits scores.

**Table 5:** Moderation analysis for the association between neuropsychological functioning and externalising problems in preadolescents, using callous traits as a moderator

	β	*se*	95% CI	*t*	*P*
Neuropsychological functioning	0.01	0.03	-0.04; 0.06	-1.31	0.190
Neuropsychological functioning x callous traits interaction	0.05	0.03	0.00; 0.10	1.87	0.062
Externalising behaviour (6)	0.29	0.03	0.23; 0.35	9.39	< 0.001**
Internalising behaviour (13)	0.40	0.03	0.34; 0.45	14.23	< 0.001**
Callousness (9)	0.19	0.03	0.12; 0.26	5.61	< 0.001**
Female sex	-0.14	0.05	-0.24; -0.04	-2.74	0.006**
Age (13)	0.03	0.02	-0.02; 0.08	1.16	0.247
Non-Dutch Western Ethnicity	-0.24	0.10	-0.43; -0.05	-2.45	0.014*
Non-Western ethnicity	-0.17	0.07	-0.30; -0.03	-2.47	0.014*
Maternal education – medium	-0.05	0.06	-0.16; 0.06	-0.83	0.407
Maternal education low	-0.07	0.19	-0.43; 0.30	-0.36	0.716
Maternal interpersonal sensitivity	-0.01	0.04	-0.09; 0.07	-0.27	0.787
Maternal depression	-0.05	0.04	-0.13; 0.03	-1.25	0.213
Maternal anxiety	-0.05	0.04	-0.12; 0.02	-1.33	0.184
Maternal hostility	0.11	0.03	0.04; 0.18	3.00	0.003**

Presented are standardised coefficients (betas) and their 95% confidence intervals

*CI* confidence interval

R^2^ = 0.51, p = < 0.001

* significant for p < 0.05, ** significant for p = < 0.01

Additional moderation analyses were executed to examine whether this result would be specific to one or more of the NEPSY domains. In the fully adjusted models, we found no significant interaction effect between callous traits and any of the neuropsychological functioning domains: attention & executive functioning (β_interaction_ = 0.04, 95% CI -0.01–0.10, *p* = 0.107), language (β_interaction_ = 0.03, 95% CI -0.03–0.08, *p* = 0.350), memory & learning (β_interaction_ = 0.05, 95% CI -0.01–0.11, *p* = 0.118), visuospatial skills (β_interaction_ = 0.02, 95% CI -0.03–0.08, *p* = 0.435), or sensorimotor skills (β_interaction_ = 0.02, 95% CI -0.04–0.08, *p* = 0.568).

In regression 4, we tested for the additional moderation effect of sex between neuropsychological functioning at age 8 and externalising problems at age 14 (Table 6). The model significantly predicted externalising behaviour in adolescents and explained 51% of the variance in externalizing behaviour at age 14 years. A main effect was found for sex (*β* = -0.14, *p* < 0.01), indicating that boys showed higher scores on externalizing behaviour than girls. However, no interaction effect for sex was found for the association between neuropsychological functioning and externalizing behaviour (β_interaction_ = 0.07, 95% CI -0.03–0.17, *p* = 0.149).

**Table 6:** Moderation analysis for the association between neuropsychological functioning and externalising problems in preadolescents, using sex as a moderator

	β	*se*	95% CI	*t*	*P*
Neuropsychological functioning	-0.02	0.03	-0.09; 0.05	-0.56	0.576
Neuropsychological functioning x sex interaction	0.07	0.05	-0.03; 0.17	1.44	0.149
Externalising behaviour (6)	0.29	0.03	0.23; 0.35	9.38	< 0.001**
Internalising behaviour (13)	0.40	0.03	0.34; 0.45	14.22	< 0.001**
Callousness (9)	0.19	0.03	0.12; 0.26	5.80	< 0.001**
Female sex	-0.14	0.05	-0.24; -0.04	-2.91	0.004**
Age (13)	0.03	0.02	-0.02; 0.08	1.13	0.257
Non-Dutch Western Ethnicity	-0.23	0.10	-0.42; -0.04	-2.42	0.016*
Non-Western ethnicity	-0.16	0.07	-0.29; -0.02	-2.31	0.021*
Maternal education – medium	-0.04	0.06	-0.16; 0.07	-0.80	0.425
Maternal education low	-0.10	0.19	-0.47; 0.27	-0.54	0.589
Maternal interpersonal sensitivity	-0.01	0.04	-0.09; 0.06	-0.39	0.696
Maternal depression	-0.06	0.04	-0.14; 0.02	-1.37	0.173
Maternal anxiety	-0.04	0.03	-0.11; 0.02	-1.27	0.205
Maternal hostility	0.11	0.04	0.04; 0.18	3.07	0.002**

Presented are standardized coefficients (betas) and their 95% confidence intervals

*CI* confidence interval

R^2^ = 0.51, p = < 0.001

* significant for p < 0.05, ** significant for p = < 0.01

When testing for differences across sex in the moderation between neuropsychological functioning and callous traits (Table 7), we did not find a three-way interaction effect (β_interaction_ = -0.02, 95% CI -0.12–0.08, *p* = 0.725). This result shows that the interaction effect among neuropsychological functioning and callous traits is the same for boys and girls.

**Table 7:** Moderation analysis for the association between neuropsychological functioning and externalising problems in preadolescents, using both callous traits and sex as moderators

	β	*se*	95% CI	*t*	*p*
Neuropsychological functioning	-0.04	0.04	-0.11; 0.03	-2.12	0.274
Neuropsychological functioning x callous traits interaction	0.07	0.03	0.01; 0.13	2.13	0.033*
Neuropsychological functioning x sex interaction	0.10	0.05	0.00; 0.20	1.35	0.057
Sex x callous traits interaction	-0.07	0.05	-0.17; 0.03	-1.28	0.201
Neuropsychological functioning x callous traits x sex interaction	-0.02	0.05	-0.12; 0.08	-0.35	0.725
Externalising behaviour (6)	0.29	0.03	0.23; 0.35	9.33	< 0.001**
Internalising behaviour (13)	0.40	0.03	0.34; 0.45	14.28	< 0.001**
Callousness (9)	0.23	0.04	0.15; 0.31	5.57	< 0.001**
Female sex	-0.14	0.05	-0.24; -0.04	-0.55	0.583
Age (13)	0.03	0.02	-0.02; 0.08	1.17	0.244
Non-Dutch Western Ethnicity	-0.25	0.10	-0.44; -0.06	-2.54	0.011*
Non-Western ethnicity	-0.16	0.07	-0.30; -0.03	-2.36	0.019*
Maternal education – medium	-0.04	0.06	-0.15; 0.07	-0.76	0.446
Maternal education low	-0.10	0.19	-0.46; 0.27	-0.52	0.603
Maternal interpersonal sensitivity	0.00	-0.04	-0.08; 0.07	-0.12	0.903
Maternal depression	-0.05	0.04	-0.13; 0.03	-0.130	0.193
Maternal anxiety	-0.04	0.03	-0.11; 0.02	-1.28	0.202
Maternal hostility	0.10	0.04	0.03; 0.17	2.82	0.005**

Presented are standardised coefficients (betas) and their 95% confidence intervals

*CI* confidence interval

R^2^ = 0.52, p = < 0.001

* significant for p < 0.05, ** significant for p = < 0.01

## Discussion

This study used data from a large, longitudinal population-based study to examine whether neuropsychological function in mid childhood is prospectively associated with externalising problems in emerging adolescence, as well as to test whether callous traits and sex moderate this association. We highlight two key findings. First, neither overall neuropsychological functioning nor its individual domains at age 8 years independently predicted later externalising problems at age 14 years over and above covariates, baseline levels of externalising problems and co-occurring internalising problems. Second, we found that callous traits at age 10 years moderated the association between neuropsychological functioning and later externalising problems to a small extent. Specifically, for children scoring low on callousness, neuropsychological functioning was not related to more externalising behaviour, while in those children who scored higher on callousness; higher neuropsychological functioning was associated with more externalising problems at age 14 years. However, this was no longer significant in fully adjusted models (i.e. once taking into account baseline levels of externalising problems and sociodemographic characteristics), suggesting that pre-existing behavioural problems are an important factor in this relationship. Future longitudinal research is needed to establish temporality and causal links. Given the use of a population-based sample with equal proportion of boys and girls, this study was also optimally suited to test for the role of sex as a potential moderator. As expected, boys showed elevated levels of callous traits and externalizing problems. However, no significant sex interactions were found in the association between neuropsychological functioning and externalising behaviour.

Overall, the current results suggest that neuropsychological functioning in childhood does not predict externalising problem behaviour in early adolescence over and above baseline levels of externalizing problems. Moreover, children with higher levels of callousness and higher neuropsychological functioning have more externalising behavioural problems than those with high neuropsychological functioning but low levels of callousness. More research will be needed to establish the aetiology of differing child neuropsychological functioning profiles in relation to externalising problems. Specific findings, implications, and future directions are discussed below.

### Neuropsychological Functioning in Mid Childhood Does Not Predict Externalising Problems in Emerging Adolescence within the General Paediatric Population

The results did not support the hypothesis that neuropsychological functioning, or its specific domains such as EF, predict later externalising problems in the general paediatric population, over and above baseline levels of externalising problems. This is not consistent with previous research that have examined the association between subdomains of neuropsychological functioning and externalising behaviour (Blanken et al., 2017; Chow et al., 2018; Petersen et al., 2015). That is, a meta-analysis found a negative relation between language ability and problem behaviour (i.e., Chow et al., 2018). However, this study used only on particular subdomain of neuropsychological functioning, while we aimed at studying the link between more broader externalizing and externalizing behaviour.

Another possible explanation for not finding a relation in our study could be, that in this study we took a more stringent approach by adjusting for multiple factors such as baseline externalising behaviour, co-occurring psychopathology, and individual neuropsychological functioning domains. To our knowledge, there is only one study (using the same sample as the current study), that took baseline externalising behaviour and co-occurring psychopathology into consideration when predicting externalising behaviour through neuropsychological functioning (Blanken et al., 2017). They did find a negative association between externalising and the attention/executive functioning domain of neuropsychological functioning specifically. However, in that study, they examined the relation between neuropsychological functioning and externalising at same age, instead of prospectively as we did in the current study. Our results highlight the importance of taking pre-existing levels of externalising problems into consideration, as this was the strongest predictor for later externalising behaviour in adolescents, consistent with other work showing that externalizing behaviour showed continuity over time (Petersen et al., 2015). It is therefore possible that previous studies would not have found neuropsychological functioning to be significantly predictive of externalising problems when they would have taking baseline externalising problems into account.

Another explanation for the null results regarding neuropsychological functioning as a predictor for externalising behaviour is the large time span between the two measurements. Neuropsychological functioning was measured at 8 years and externalising behaviour at 14 years, corresponding with a mean time span of 5 years between measurements. Studies that did find evidence for neuropsychological functioning as a predictor for later externalising behaviour used a shorter time interval between measurement. For example, in the study of Blanken et al. (2017), the mean time span between two measurements (subdomains of neuropsychological functioning and externalising problems) was 1.85 years. Thompson et al. (2019) measured the two constructs at approximately the same time.

Ultimately, the current results also show that the lack of finding a main affect for neuropsychological functioning might be explained by the moderation of callous traits. That is, the different directions of associations between neuropsychological functioning and externalising depending on the level of callousness, cancel each other out when examining neuropsychological functioning in the whole sample, not stratified by these levels of callousness.

### Callousness Moderates the Association between Neuropsychological Functioning and Externalising Problems

Our hypotheses that callousness would predict later externalising behaviour and would moderate the relationship between neuropsychological functioning, and externalising behaviour were partially confirmed. Children with higher scores on callousness and higher neuropsychological functioning had more externalising problems, whereas no association between neuropsychological functioning and externalising problems was found for children with low levels of neuropsychological functioning. It should be borne in mind that this interaction effect was small and dropped below the statistical significance level of *P*-value = 0.05 when adjusting for confounders, although confidence intervals were overlapping between the non-adjusted and fully-adjusted interaction estimates.

These results are in line with other studies that showed an opposite pattern of associations between neuropsychological functioning and externalising problems based on their levels of callousness (Baskin-Sommers et al., 2015; Dotterer et al., 2021), and also adds to these previous findings in several ways. For instance, Baskin-Sommers et al. (2015) found a divergent relation between executive functions and earlier externalizing problems in predicting violence and substance use in a high-risk sample, while we found this nominally divergent pattern when looking at neuropsychological functioning’to predict externalizing problems in a prospective population-based cohort of child development.

Additionally, although Dotterer et al. (2021) studied intelligence and several aspects of executive functioning, they only found an interaction for CU-traits in sustained attention and antisocial behaviour. However, again, in our sample we additionally found an interaction for overall neuropsychological functioning in a prospective way. The difference might also lie in the fact that this study, as in the Baskin-Sommers et al. (2015) study, the sample was oversampled for children in poverty, and thus high-risk.

Our current findings add to the existing literature by presenting the extended small moderating effect of callous traits in the association between neuropsychological functioning and externalising problems, and in a prospective design.

### No Moderation of Sex

Based on previous literature, we expected boys to show more callous traits and externalising, but we did not formulate a priori hypotheses regarding sex as a moderator, given that previous studies have focused mainly on males. Current results did show higher levels of externalising and callous traits in males compared to females, but no moderation of sex on the association between neuropsychological functioning and externalising in later adolescence. Although the higher levels of externalising problems and interpersonal callousness in boys was expected and in line with previous findings (e.g., Essau et al., 2006; Mayes et al., 2020; Pihet et al., 2015), the fact that we did not find effects of sex on neuropsychological function is not in line with studies finding sex effects in neuroimaging studies on CU-traits (e.g. Rogers et al., 2019; Villemonteix et al., 2022).

### Limitations and Future Directions

One limitation of the current research is that most participants have the Dutch nationality; consequently, results may not generalise to other national backgrounds.

Another limitation is the use of the shortened NEPSY-II-NL to measure neuropsychological functioning. Due to time constraints, only a few tasks were administered, which means not all neuropsychological constructs are thoroughly measured. Nevertheless, this short version has shown to correlate highly with the total and taps domains of neuropsychological functioning that are previously implicated in externalising psychopathology (see White et al., 2018).

Additionally, the CBCL and interpersonal callousness were measured through maternal reports and not by child reports, which might have led to biased data (Najman et al., 2001). For example, some authors suggest that mothers with elevated psychopathology are more easily distressed by their child’s problem behaviour, resulting in distorted and higher scores on their child’s problems (De Los Reyes & Kazdin, 2005). Therefore, we have adjusted for maternal psychopathology. Furthermore, neuropsychological functioning was objectively assessed by trained researchers.

A last limitation might be that callousness was assessed at age 10 years, so in terms of timing between both predictor (neuropsychological functioning at age 8 years), and outcome measure (i.e. externalising at age 14 years), therefore it was not possible to account for baseline callousness. Consequently, it is unknown what the unique influence is of callousness, over and above pre-existing callousness. Similarly, although externalizing was controlled for at age 6 years, this was not done for age 8 years because there was no data available to include (i.e. was not assessed at that time point). For these reasons, it is recommended to replicate this research, while accounting for baseline callousness.

Last, missing value analyses revealed important differences in neuropsychological function between the excluded participants and included participants. Results showed that excluded participants scored significantly lower on different domains of neuropsychological functioning and covariates. This may have influenced our results. However, multiple imputation was applied to the confounders in order to adjust for potential missingness bias in our analyses.

### Implications and Future Research

Children with externalizing problem behaviour are at risk for negative individual functioning later in life. The present findings highlight the need to differentiate between children with externalising problems who also have high levels of callousness and those with low callousness, as they may present with different neuropsychological profiles. Additionally, this differentiation in children and adolescents with problem behaviour might also result in different types of externalizing behaviour. Although not studied here, it might be interesting to study whether children with lower neuropsychological functioning and lower callous traits show other types of externalizing behaviours, such as reactive forms of aggression, while those children with higher neuropsychological functioning and high callousness are inclined to show more proactive forms of violence and cruel behaviour (Rosa-Justicia et al., 2020). Although the current study added on evidence suggesting the delineation of two subgroups by examining associations between neuropsychological functioning, externalising behaviour and callousness prospectively, the mechanisms of this interactive relation remain unclear. Causal relations might need to be further established using causal inference methods.

Current findings also support new directions in clinical practice, where a distinction in different groups of externalizing problem behaviour characterized by high versus low levels of CU- traits has recently been adopted by the DSM-5 through the inclusion of a ‘limited-prosocial emotions’ specifier for CD, with these two subgroups showing distinct profiles in terms of aetiology, behavioural outcomes and neurocognition (Vanwoerden et al., 2016; Viding & McCrory, 2018).

This specification of children with conduct problems and differing levels of CU-traits has also implications for treatment of these problems (Andrade et al., 2022). That is, since they show different core dysfunctions underlying their behaviour, treatment must match with these different core dysfunctions by either focusing more on the low neuropsychological functioning or focus more on the callous traits. However, more research is needed to determine which treatment intervention works best for children displaying externalising behaviour with varying levels of neuropsychological functioning, and for those with and without CU-traits (Bakker et al., 2017).

### Conclusion

In sum, the current findings do not show support for the association between child neuropsychological functioning and externalising behaviour in preadolescence within the general population. However, when stratifying by callous traits, results indicate that neuropsychological functioning does associate with externalising problems, but in differential directions depending on levels of callousness. That is, in children who display higher levels of callousness, higher neuropsychological functioning relates to externalising behaviour in later adolescence, whereas in those with lower levels of callousness, no association was found. This study also highlights the importance of child-onset problem behaviour and how this predicts later externalising problems in adolescence. Treatment efforts should be directed at preventing externalising problems at a young age, with the emphasis on children who display high CU-traits. Research has shown that if interventions are tailored fitting the unique emotional, cognitive, and motivational profile of children with CU-traits, behavioural problems can be reduced (Frick et al., 2014). Future studies should look further into the underlying neurocognitive mechanisms of why elevated CU-traits and high neuropsychological functioning lead to more externalising behaviour and aggression in later life.

### Supplementary Information

Below is the link to the electronic supplementary material.

Supplementary file1 (DOCX 33 KB)

## Data Availability

The data that support the findings of this study can be obtained upon request. Requests should be directed to the management team of the Generation R Study (secretariaat.genr@erasmusmc.nl), which has a protocol for approving data requests. Because of restrictions based on privacy regulations and informed consent of participants, data cannot be made freely available in a public repository.
