# Supplementary material for: Child Neuropsychological Functioning and Interpersonal Callousness as Predictors of Externalising Behaviour in Early Adolescence: A Prospective Population-based Study
Source: Res Child Adolesc Psychopathol. 2023 Jun 8;51(10):1465–80. doi: 10.1007/s10802-023-01091-8 (PMC10543790; doi:10.1007/s10802-023-01091-8)
Supplement: Supplementary file 1 — Supplementary file1 (DOCX 33 KB) [file 10802_2023_1091_MOESM1_ESM.docx]

**Supplemental Tables & Figures paper executive functioning and externalising problems**

Table S1: Non-imputed regression analysis for the association between neuropsychological functioning and subsequent externalising problems in preadolescents.

|  | β | *se* | 95% CI | *t* | *P* |
| --- | --- | --- | --- | --- | --- |
| Neuropsychological functioning | -0.01 | 0.05 | -0.06; 0.05 | -0.17 | 0.869 |
| Externalising behaviour (6) | 0.31 | 0.03 | 0.25; 0.38 | 9.41 | <0.001** |
| Internalising behaviour (13) | 0.39 | 0.03 | 0.33; 0.45 | 12.34 | <0.001** |
| Callousness (9) | 0.18 | 0.03 | 0.12; 0.24 | 5.75 | <0.001** |
| Female sex | -0.16 | 0.06 | -0.27; -0.05 | -2.83 | 0.005** |
| Age (13) | 0.03 | 0.03 | -0.02; 0.09 | 1.26 | 0.210 |
| Non-Dutch Western Ethnicity | -0.22 | 0.11 | -0.44; 0.00 | -1.97 | 0.049* |
| Non-Western ethnicity | -0.09 | 0.08 | -0.24; 0.08 | -1.05 | 0.293 |
| Maternal education – medium | -0.06 | 0.06 | -0.18; 0.06 | -0.98 | 0.326 |
| Maternal education low | -0.16 | 0.21 | -0.57; 0.26 | -0.74 | 0.459 |
| Maternal interpersonal sensitivity | -0.09 | 0.04 | -0.17; -0.01 | -2.30 | 0.022* |
| Maternal depression | -0.02 | 0.04 | -0.11; 0.07 | -0.42 | 0.677 |
| Maternal anxiety | 0.00 | 0.04 | -0.07; 0.07 | -0.08 | 0.936 |
| Maternal hostility | 0.14 | 0.04 | 0.06; 0.22 | 3.57 | <0.001** |

Note: R^2^ = 0.51, p = < .001. * significant for p < .05, ** significant for p = < .01. CI = confidence interval. Presented are standardised coefficients (betas) and their 95% confidence intervals.

Table S2: Non-imputed multiple regression analysis for the association between the subdomains of neuropsychological functioning and externalising problems in preadolescents.

|  | β | *se* | 95% CI | *t* | *P* |
| --- | --- | --- | --- | --- | --- |
| Attention and executive functioning | -0.02 | 0.05 | -0.08; 0.05 | -0.56 | 0.573 |
| Language | 0.01 | 0.03 | -0.06; 0.07 | 0.23 | 0.820 |
| Memory and learning | 0.05 | 0.03 | -0.02; 0.12 | 1.35 | 0.178 |
| Visuo-spatial functioning | -0.04 | 0.04 | -0.11; 0.03 | -1.06 | 0.289 |
| Sensorimotor functioning | -0.01 | 0.04 | -0.09; 0.06 | -0.40 | 0.689 |
| Externalising behaviour (6) | 0.31 | 0.03 | 0.25; 0.38 | 9.34 | <0.001** |
| Internalising behaviour (13) | 0.39 | 0.03 | 0.33; 0.45 | 12.37 | <0.001** |
| Callousness (9) | 0.18 | 0.03 | 0.12; 0.24 | 5.67 | <0.001** |
| Female sex | -0.18 | 0.06 | -0.29; -0.06 | -2.91 | 0.004** |
| Age (13) | 0.04 | 0.03 | -0.02; 0.09 | 1.24 | 0.217 |
| Non-Dutch Western Ethnicity | -0.23 | 0.11 | -0.44; -0.01 | -2.06 | 0.040* |
| Non-Western ethnicity | -0.09 | 0.08 | -0.25; 0.07 | -1.08 | 0.280 |
| Maternal education – medium | -0.06 | 0.06 | -0.18; 0.06 | -1.02 | 0.307 |
| Maternal education low | -0.17 | 0.21 | -0.59; 0.24 | -0.82 | 0.415 |
| Maternal interpersonal sensitivity | -0.09 | 0.04 | -0.17; -0.01 | -2.20 | 0.029* |
| Maternal depression | -0.03 | 0.04 | -0.12; 0.06 | -0.62 | 0.533 |
| Maternal anxiety | 0.00 | 0.04 | -0.07; 0.07 | -0.02 | 0.986 |
| Maternal hostility | 0.14 | 0.04 | 0.06; 0.22 | 3.57 | <0.001** |

Note: R^2^ = 0.51, p = < .001. * significant for p < .05, ** significant for p = < .01. CI = confidence interval. Presented are standardised coefficients (betas) and their 95% confidence intervals.

Table S3: Non-imputed moderation analysis for the association between neuropsychological functioning and externalising problems in preadolescents, using callous traits as a moderator.

|  | β | *se* | 95% CI | *t* | *P* |
| --- | --- | --- | --- | --- | --- |
| Neuropsychological functioning | -0.01 | 0.03 | -0.07; 0.05 | -0.22 | 0.825 |
| Neuropsychological functioning x callous traits interaction | 0.06 | 0.03 | 0.01; 0.12 | 2.25 | 0.02* |
| Externalising behaviour (6) | 0.31 | 0.03 | 0.25; 0.38 | 9.38 | <0.001** |
| Internalising behaviour (13) | 0.39 | 0.03 | 0.32; 0.45 | 12.31 | <0.001** |
| Callousness (9) | 0.18 | 0.03 | 0.12; 0.24 | 5.74 | <0.001** |
| Female sex | -0.15 | 0.06 | -0.27; -0.04 | -2.72 | 0.007** |
| Age (13) | 0.04 | 0.03 | -0.02; 0.09 | 1.25 | 0.211 |
| Non-Dutch Western Ethnicity | -0.23 | 0.11 | -0.45; -0.01 | -2.09 | 0.037* |
| Non-Western ethnicity | -0.09 | 0.08 | -0.25; 0.08 | -1.05 | 0.292 |
| Maternal education – medium | -0.06 | 0.06 | -0.17; 0.06 | -0.92 | 0.360 |
| Maternal education low | -0.12 | 0.21 | -0.53; 0.30 | -0.55 | 0.586 |
| Maternal interpersonal sensitivity | -0.08 | 0.04 | -0.16; 0.00 | -2.02 | 0.043* |
| Maternal depression | -0.01 | 0.04 | -0.10; 0.07 | -0.29 | 0.770 |
| Maternal anxiety | -0.01 | 0.04 | -0.08; 0.06 | -0.23 | 0.820 |
| Maternal hostility | 0.13 | 0.04 | 0.06; 0.21 | 3.37 | 0.001** |

Note: R^2^ = 0.51, p = < .001. * significant for p < .05, ** significant for p = < .01. CI = confidence interval. Presented are standardised coefficients (betas) and their 95% confidence intervals.

Table S4: Non-imputed moderation analysis for the association between neuropsychological functioning and externalising problems in preadolescents, using sex as a moderator.

|  | β | *se* | 95% CI | *t* | *P* |
| --- | --- | --- | --- | --- | --- |
| Neuropsychological functioning | -0.03 | 0.04 | -0.11; 0.05 | -0.73 | 0.467 |
| Neuropsychological functioning x sex interaction | 0.05 | 0.06 | -0.06; 0.17 | 0.90 | 0.370 |
| Externalising behaviour (6) | 0.31 | 0.03 | 0.25; 0.38 | 9.37 | <0.001** |
| Internalising behaviour (13) | 0.39 | 0.03 | 0.33; 0.45 | 12.36 | <0.001** |
| Callousness (9) | 0.18 | 0.03 | 0.12; 0.24 | 5.79 | <0.001** |
| Female sex | -0.17 | 0.06 | -0.28; -0.05 | -2.90 | 0.004** |
| Age (13) | 0.04 | 0.03 | -0.02;0.09 | 1.28 | 0.203 |
| Non-Dutch Western Ethnicity | -0.22 | 0.11 | -0.44; 0.00 | -2.00 | 0.046* |
| Non-Western ethnicity | -0.08 | 0.08 | -0.25; 0.08 | -1.01 | 0.314 |
| Maternal education – medium | -0.06 | 0.06 | -0.18; 0.06 | -0.96 | 0.336 |
| Maternal education low | -0.18 | 0.21 | -0.60; 0.24 | -0.85 | 0.395 |
| Maternal interpersonal sensitivity | -0.09 | 0.04 | -0.17; -0.01 | -2.27 | 0.024* |
| Maternal depression | -0.02 | 0.04 | -0.10; 0.07 | -0.41 | 0.679 |
| Maternal anxiety | 0.00 | 0.04 | -0.08; 0.07 | -0.09 | 0.926 |
| Maternal hostility | 0.14 | 0.04 | 0.06; 0.22 | 3.49 | <0.001** |

Note: R^2^ = 0.51, p = < .001. * significant for p < .05, ** significant for p = < .01. CI = confidence interval. Presented are standardised coefficients (betas) and their 95% confidence intervals.

Table S5: Non-imputed moderation analysis for the association between neuropsychological functioning and externalising problems in preadolescents, using both callous traits and sex as moderators.

|  | β | *se* | 95% CI | *t* | *p* |
| --- | --- | --- | --- | --- | --- |
| Neuropsychological functioning | -0.06 | 0.04 | -0.14; 0.03 | -1.33 | 0.184 |
| Neuropsychological functioning x callous traits interaction | 0.09 | 0.04 | 0.02; 0.17 | 2.56 | 0.011* |
| Neuropsychological functioning x sex interaction | 0.09 | 0.06 | -0.03; 0.21 | 1.45 | 0.146 |
| Sex x callous traits interaction | -0.06 | 0.06 | -0.17; 0.05 | -1.05 | 0.294 |
| Neuropsychological functioning x callous traits x sex interaction | -0.05 | 0.06 | -0.17; 0.06 | -0.91 | 0.361 |
| Externalising behaviour (6) | 0.31 | 0.03 | 0.25; 0.38 | 9.31 | <0.001** |
| Internalising behaviour (13) | 0.39 | 0.03 | 0.33; 0.45 | 12.33 | <0.001** |
| Callousness (9) | 0.21 | 0.04 | 0.13; 0.30 | 4.97 | <0.001** |
| Female sex | -0.16 | 0.06 | -0.28; -0.05 | -2.84 | 0.005** |
| Age (13) | 0.04 | 0.03 | -0.02; 0.09 | 1.28 | 0.201 |
| Non-Dutch Western Ethnicity | -0.24 | 0.11 | -0.46; -0.02 | -2.15 | 0.032* |
| Non-Western ethnicity | -0.09 | 0.08 | -0.25; 0.08 | -1.04 | 0.301 |
| Maternal education – medium | -0.05 | 0.06 | -0.58; 0.26 | -0.90 | 0.371 |
| Maternal education low | -0.16 | 0.21 | -0.15; 0.00 | -0.73 | 0.463 |
| Maternal interpersonal sensitivity | -0.07 | 0.04 | -0.15; 0.00 | -1.85 | 0.064 |
| Maternal depression | -0.01 | 0.04 | -0.10; 0.07 | -0.31 | 0.754 |
| Maternal anxiety | -0.01 | 0.04 | -0.08; 0.06 | -0.20 | 0.839 |
| Maternal hostility | 0.13 | 0.04 | 0.05; 0.21 | 3.17 | 0.002** |

Note: R^2^ = 0.52, p = < .001. * significant for p < .05, ** significant for p = < .01. CI = confidence interval. Presented are standardised coefficients (betas) and their 95% confidence intervals.
